# Three-Dimensional Speckle-Tracking Echocardiography-Derived Left Ventricular Global Longitudinal Strain and Mitral Annular Plane Systolic Excursion Are Associated in Healthy Adults—Insights from the MAGYAR-Healthy Study

**DOI:** 10.3390/biomedicines13030625

**Published:** 2025-03-04

**Authors:** Attila Nemes, Nóra Ambrus, Csaba Lengyel

**Affiliations:** Department of Medicine, Albert Szent-Györgyi Medical School, University of Szeged, P.O. Box 427, 6725 Szeged, Hungary; ambrusnora@gmail.com (N.A.); lecs@in1st.szote.u-szeged.hu (C.L.)

**Keywords:** left ventricular, mitral annular plane systolic excursion, strain, three-dimensional echocardiography, healthy

## Abstract

**Introduction:** Mitral annular plane systolic excursion (MAPSE) is a quantitative feature of left ventricular (LV) function that can be easily measured by M-mode echocardiography. The more recent LV strains represent LV contractility, and they can be determined for the three main directions in the radial (RS), longitudinal (LS), and circumferential (CS) directions. Three-dimensional (3D) speckle-tracking echocardiography (3DSTE) seems to be ideal for the simultaneous assessment of all LV strains from the same acquired 3D dataset. Their associations, however, have not been fully analyzed in clinical settings in healthy volunteers yet. Therefore, the present study aimed to find associations between MAPSE and 3DSTE-derived LV strains in healthy circumstances. **Methods:** The present study consisted of 106 healthy adult volunteers (mean age 28.1 ± 6.3 years, 48 men). Complete two-dimensional Doppler echocardiography with MAPSE assessment and 3DSTE-derived LV strain measurements were performed in all cases. **Results:** Tendentiously reduced LV volumes with preserved LV ejection fraction were present in the case of mean MAPSE as compared to decreased/increased MAPSE. Global and regional LV-RS and LV-CS did not differ if MAPSE was increased, mean or decreased. Global LV-LS was reduced in the case of decreased MAPSE, mainly due to tendentiously higher apical LV-LS. MAPSE did not differ if global LV-RS and LV-CS were increased, mean or decreased. MAPSE was tendentiously increased in the presence of increased global LV-LS. **Conclusions:** Only 3DSTE-derived global LV-LS and MAPSE are associated in healthy adults; LV global strains in other directions show no relationship with MAPSE.

## 1. Introduction

In recent decades, echocardiography has undergone enormous technical development. While old well-established methods have remained in clinical practice, new ones have spread rapidly and become everyday practice. There are several echocardiographic parameters used to characterize left ventricular (LV) systolic function [1]. The up-and-down motion of the mitral annular (MA) plane during the cardiac cycle is a quantitative feature of LV longitudinal function that can be measured using M-mode echocardiography (MME) [2,3,4,5,6,7]. The method is simple, easy to implement, and validated, and the parameter is called MA plane systolic excursion (MAPSE). In other words, MAPSE refers to LV longitudinal shortening, MA motion, left atrioventricular plane displacement, or mitral ring displacement, which can be affected earlier than LV ejection fraction (EF). MAPSE is known for its significant prognostic power as well [2,3,4,5,6,7]. The more recent LV strains as quantitative representatives of LV contractility are to be determined by echocardiography as well in the three main directions of the space in the radial (RS), longitudinal (LS), and circumferential (CS) directions [8]. Three-dimensional (3D) speckle-tracking echocardiography (STE) seems to be ideal for the simultaneous assessment of LV strains using virtually created LV models from the same acquired 3D dataset [9,10,11,12]. On the one hand, it may seem that partially similar and interchangeable parameters can be calculated with both methods. On the other hand, both methods have their own advantages and disadvantages. Furthermore, the associations between MAPSE and 3DSTE-derived strains have not been fully analyzed in clinical settings even in healthy volunteers yet. Therefore, the present study aimed to find associations between MAPSE as assessed by MME and 3DSTE-derived LV strains in healthy circumstances. It was also examined whether the relationship between these parameters shows differences depending on whether they are average or smaller/larger.

## 2. Materials and Methods

Subjects. The present study consisted of 106 healthy adult volunteers (mean age: 28.1 ± 6.3 years, 48 men) who were involved in the present study between 2011 and 2017. All participants were considered to be healthy due to the fact that their medical history did not contain any known disorder or other condition that could theoretically affect the findings. No one was obese, a professional athlete, pregnant, or a smoker at the time of enrollment. In all cases, physical examination, a laboratory test, electrocardiography (ECG), and two-dimensional (2D) Doppler echocardiography were performed with a negative result. 3DSTE was performed at the time of the enrollment with 3D echocardiographic data acquisition, and their analysis was performed at a later date. The present retrospective study is an analysis from the ‘Motion Analysis of the heart and Great vessels bY three-dimensionAl speckle-tRacking echocardiography in Healthy subjects’ (MAGYAR-Healthy) Study. This study was partly organized for physiologic studies to compare 3DSTE-derived parameters in healthy individuals among others (‘Magyar’ means ‘Hungarian’ in the Hungarian language). This study was performed in accordance with the Helsinki Declaration (revised in 2013); it was approved by the Institutional and Regional Biomedical Research Committee of the University of Szeged (number 71/2011), and informed consent was given by all participants.

**Figure 1:**
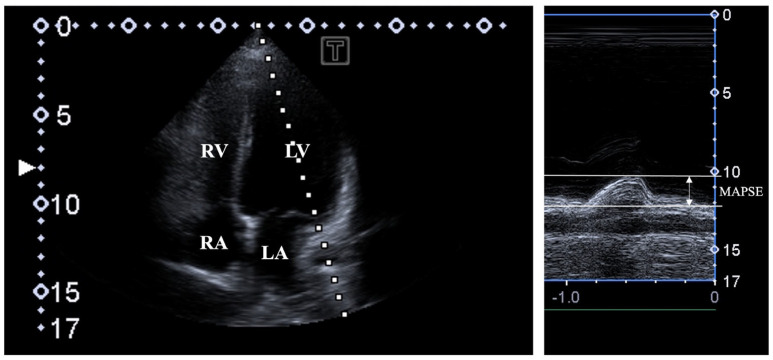
Assessment of mitral annular plane systolic excursion (MAPSE) by M-mode echocardiography in apical four-chamber view. Abbreviations: LA = left atrium; LV = left ventricle; RA = right atrium; RV = right ventricle; MAPSE = mitral annular plane systolic excursion.

Two-dimensional Doppler echocardiography. A Toshiba Artida^TM^ cardiac ultrasound device (Toshiba Medical Systems, Tokyo, Japan) was used in all tests attached to a PST-30BT (1–5 MHz) phased-array transducer. In all individuals being in the left lateral decubitus position, the transducer was placed on their chest in typical parasternal and apical positions by the observer. In all cases, LA and LV were quantified, and Simpsons’s LV-EF measurement was carried out. Significant valvular stenosis and regurgitation were excluded by Doppler echocardiography. LV diastolic function was assessed by measuring transmitral flow E and A velocities and their ratio by pulsed Doppler. MAPSE was measured in the apical long-axis view as the movement of the lateral MA edge towards the apex of the LV in systole by MME (Figure 1) [2].

**Figure 2:**
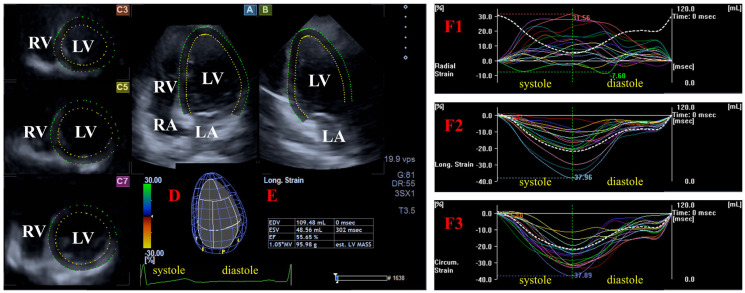
Assessment of the left ventricular (LV) strains by three-dimensional (3D) speckle-tracking echocardiography. Apical four-chamber (A) and two-chamber (B) long-axis views and short-axis views at the basal (C3), midventricular (C5), and apical levels (C7) of the LV are presented together with a 3D cast of the LV (D) and the LV volumetric data calculated (E). Curves of time—global and segmental radial (F1), longitudinal (F2), and circumferential (F3) LV strains (white and colored lines) and time—change in the LV volume (dashed white line) during the cardiac cycle are shown together. Abbreviations: LA = left atrium; LV = left ventricle; RA = right atrium; RV = right ventricle; EDV = end-diastolic volume; ESV = end-systolic volume; EF = ejection fraction.

Three-dimensional speckle-tracking echocardiography. The same echocardiographic tool was used for 3DSTE after changing the transducer to a PST-25SX matrix phased-array transducer [9,10,11,12]. Then, 3D echocardiographic datasets were acquired from the apical window. To reach optimal image quality, 6 subvolumes within 6 cardiac cycles during a breathhold were acquired. Version 2.7 of 3D Wall Motion Tracking software (Ultra Extend, Toshiba Medical Systems, Tokyo, Japan) was used for the analysis of the auto-created merged 3D full-volume dataset. Apical four-chamber (AP4CH) and two-chamber (AP2CH) long-axis views and 3 cross-sectional views were automatically created, and then septal and lateral edges of the LV-MA and the endocardial surface of the LV apex were determined by the observer. Following automatic contour detection and sequential analysis, a virtual 3D model of the LV was created, and the following global and basal, midventricular, and apical regional unidirectional/unidimensional LV strains were calculated as a mean of segmental ones (Figure 2) [1,2,8,9,10,11,12]:

- Radial (RS) representing the thickening/thinning of the LV;
- Circumferential (CS) representing the narrowing/widening of the LV;
- Longitudinal strain (LS) representing the shortening/lengthening of the LV.

Statistical analysis. All continuous variables were represented in mean ± standard deviation (SD) format together with the median (in bracket). Statistical significance was considered to be present in the case of a p of less than 0.05. Data were analyzed by the analysis of variance (ANOVA) test for normal distribution data and the Kruskal–Wallis H test for non-normal distribution data. Pearson’s correlation coefficients were calculated between MAPSE and global and regional LV strains. Non-parametric tests alongside parametric methods were used where appropriate (e.g., Spearman rank correlation). Correlations between MAPSE and the global and regional LV strain were assessed using linear regression analysis as well. SPSS software version 22 (SPSS Inc., Chicago, IL, USA) was used for statistical analyses.

## 3. Results

Clinical data. Routine clinical data including systolic and diastolic blood pressures (122.3 ± 3.2 mmHg and 83.3 ± 2.1 mmHg, respectively), heart rate (71.0 ± 1.8 1/s), height (169.3 ± 9.8 cm), weight (72.9 ± 14.3 kg), and body surface area (1.84 ± 0.33 m^2^) were in normal ranges in all healthy subjects.

Two-dimensional Doppler echocardiography. Echocardiographic measures including LA diameter (37.2 ± 3.6 mm), LV end-diastolic diameter and volume (48.0 ± 3.6 mm and 106.7 ± 22.7 mL, respectively), LV end-systolic diameter and volume (32.1 ± 3.2 mm and 37.9 ± 9.2 mL, respectively), interventricular septum and LV posterior wall (9.1 ± 1.2 mm and 9.4 ± 1.5 mm, respectively), LV-EF (64.5 ± 3.7%), and early and late mitral inflow velocities (78.8 ± 16.7 and 59.3 ± 14.5 cm, respectively) were within the normal reference ranges. Larger than grade 1 valvular regurgitation or significant valvular stenosis could not be found in case of any valves in any cases.

Classification of subjects. The group of healthy subjects was classified according to the following: mean ± SD of MAPSE, global LV-RS, LV-CS, and LV-LS were calculated, and then three subgroups were created based on the lower (11 mm, 15.7%, −22.5%, and −13.9%, respectively) and upper (17 mm, 35.5%, −32.1%, and −18.7%, respectively) values of these parameters.

**Figure 3:**
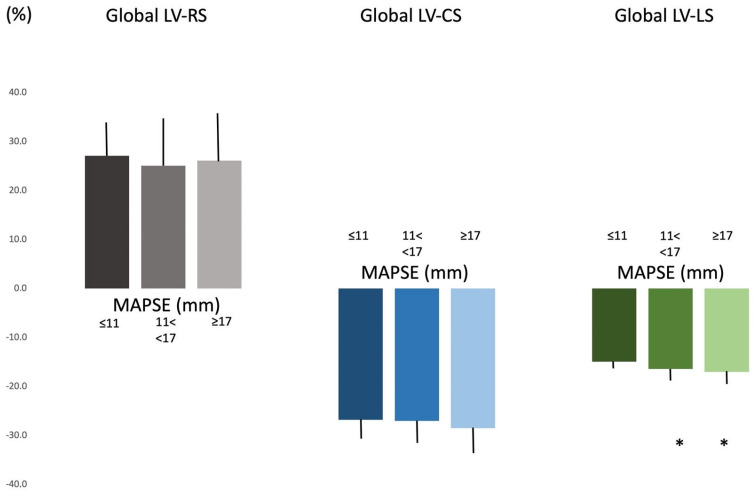
Left ventricular global strains in different mitral annular plane systolic excursion groups. * *p* < 0.05 vs. MAPSE ≤ 11 mm. Abbreviations: LV = left ventricular, RS = radial strain, CS = circumferential strain, LS = longitudinal strain, MAPSE = mitral annular plane systolic excursion.

LV strains in MAPSE subgroups. Tendentiously reduced LV volumes were present in the case of mean MAPSE as compared to decreased/increased MAPSE. Global and regional LV-RS and LV-CS did not differ in the MAPSE subgroups. Global LV-LS was reduced in case of decreased MAPSE, mainly due to tendentiously higher apical LV-LS (Table 1, Figure 3).

**Figure 4:**
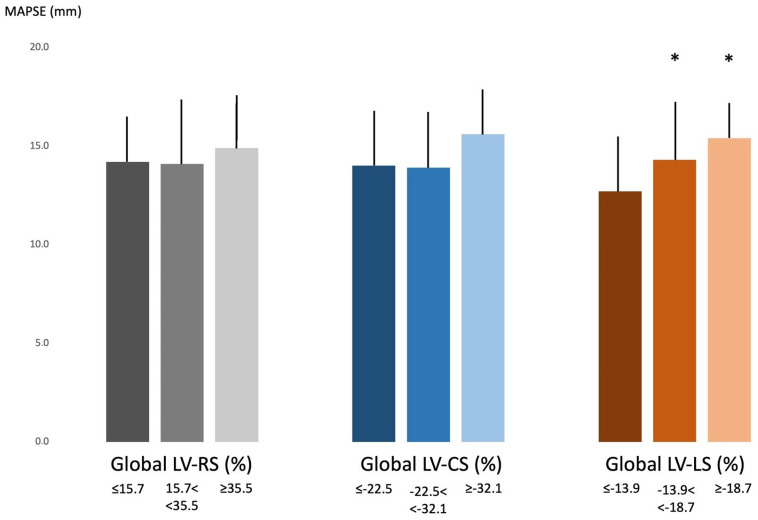
Mitral annular plane systolic excursion in different left ventricular global strain groups. * *p* < 0.05 vs. global LV-LS ≤ −13.9%. Abbreviations: LV = left ventricular, RS = radial strain, CS = circumferential strain, LS = longitudinal strain, MAPSE = mitral annular plane systolic excursion.

MAPSE in LV strain subgroups. With increasing global LV-RS, a parallel increase in LV-CS and preserved global LV-LS could be detected with the highest LV end-diastolic volume and LV-EF in the case of the highest global LV-RS. With increasing global LV-CS, a parallel increase in LV-RS could be detected with the lowest LV end-systolic volume and the highest LV-EF and global LV-LS at the highest global LV-CS. With increasing global LV-LS, global LV-RS remained unchanged, and LV end-systolic volume was the lowest, while LV-EF and global LV-CS were the highest in the case of the highest global LV-LS. MAPSE did not differ in the global LV-RS and LV-CS subgroups. MAPSE was increased in case of increased global LV-LS (Table 2, Figure 4).

Correlation and regression analyses. MAPSE did not show correlations with global (r = 0.04, *p* = 0.66), basal (r = 0.01, *p* = 0.91), midventricular (r = 0.07, *p* = 0.45), and apical (r = 0.07, *p* = 0.49) LV-RS, global (*p* = −0.16, *p* = 0.09), basal (r = 0.09, *p* = 0.37), midventricular (r = −0.15, *p* = 0.12), and apical (r = −0.21, *p* = 0.07) LV-CS, and basal (r = −0.14, *p* = 0.16), midventricular (*p* = −0.10, *p* = 0.31), and apical (r = −0.18, *p* = 0.07) LV-LS. Only global LV-LS (r = −0.27, *p* = 0.004) showed mild correlations with MAPSE. Linear regression analysis revealed correlations only between global LV-LS and MAPSE (r = −0.35, *p* = 0.009).

## 4. Discussion

In clinical practice, a number of echocardiographic parameters are used to assess the systolic function of the LV in addition to the LV-EF [1]. One of the simplest MME-based parameters is MAPSE, which is defined as the displacement of the lateral MA edge respecting the cardiac cycle [2,3]. MAPSE is widely used in different clinical scenarios and validated, and its significant prognostic power has also been confirmed [2,3,4,5,6,7,13].

Echocardiography has undergone enormous development in recent decades. One of the most important results of this process is the appearance of STE and the resulting strains being increasingly used in clinical practice [14]. If the strain is defined in a loop fixed in a given plane, then it is 2DSTE, if it is defined in an acquired 3D echocardiographic database, it is called 3DSTE [9,10,11,12,14]. The latter one is considered to be the most modern echocardiographic technique since it is able to determine strains in predefined radial (RS), longitudinal (LS), and circumferential (CS) directions simultaneously from a given acquired 3D dataset. Moreover, LV strains featuring the whole LV (global) and its segments (segmental) can be calculated at the same time together with derived regional and mean segmental LV strains [9,10,11,12].

The saddle-shaped MA has a fibrotic structure with a dynamic motion throughout the cardiac cycle; its spatial movement depends largely on the musculature of the adjacent heart cavities, like that of LV [15]. Due to the above facts, MAPSE is a functional feature of LV like the strains, which can excellently characterize the spatial contractility of the LV [1,2,3,4,5,6,7]. 3DSTE is validated for LV volumetric and strain assessments [16,17,18,19]. In recent 3DSTE studies from the MAGYAR-Healthy Study, observations confirmed several associations between LV volumes and strains after the determination of their normal reference values [20,21,22]. Therefore, the aim of the present study was to confirm associations between MAPSE and LV strains under healthy conditions by also examining what happens when parameters are lower or larger than the average.

In a previous paper from the MAGYAR-Healthy Study, a similar parameter was measured for the right ventricle (RV), which was calculated for the tricuspid annulus (TA), called TA plane systolic excursion (TAPSE), characterizing the longitudinal function of the RV. Although MAPSE and TAPSE are calculated in the same way, LV and RV are ventricles with different shapes and functional properties, which can partially explain differences in sample size due to exclusions in these studies [23]. Regarding the results of the present study, the expected findings have confirmed that global LV-LS representing longitudinal LV contraction and MAPSE are associated with healthy adults. These results were primarily due to apical longitudinal contraction, represented by (non-significant) changes in regional apical LV-LS. However, it has also been shown that strains representing systolic radial thickening (global LV-RS) and circumferential narrowing (global LV-CS) show no associations with MAPSE. These findings suggest differences between MAPSE and TAPSE; the latter showed associations with global LV-RS in healthy adults [23].

The presented results have several implications. First, although 3DSTE-derived LV-LS and MAPSE are similar features of LV longitudinal function, they are fundamentally different [3,4,5,9,10,11,12]. MAPSE is not only able to assess the LV regional systolic function but also the global LV longitudinal function [4]. Specific LV strains are more complex and seem to be more relevant characteristics of LV contractility in all directions of the space [8,9,10,11,12]. However, LV strain assessment is highly dependent on the operator’s experience [24], on the frame rate setting [25], and on the chest wall conformation [26]. For instance, a narrow antero-posterior thoracic diameter may cause an increased intra- and inter-rater reliability in LV-LS assessment [26]. Nevertheless, MAPSE can be successfully used clinically because it can be easily determined with MME [2,3,4]. Secondly, MAPSE shows associations only with LV-LS; other LV strains have no relationship with it. These findings were expected but could be quantitatively confirmed in the present study. Thirdly, although only tendentious differences could be confirmed, regional apical LV-LS was associated with MAPSE, not regional basal LV-LS. These findings can indicate the importance that associations should be examined in certain pathologies to see what happens in case of the presence of subclinical LV abnormalities. This could be a topic of future investigations, which could help understand the development of early-stage heart failure.

Limitation section. The most important limitations that arose during the study are listed below.

- The most important technical limitation associated with 3DSTE is its lower image quality compared to 2D echocardiography, which may have affected the findings [5,6,7,8].
- Although 3DSTE-capable software offers simultaneous assessment of LV rotational parameters, the present study did not aim to determine them due to their complexity. This could be a topic of future investigations.
- 3DSTE offers complex 3D analysis of all other chambers, but analyzing the results would have gone significantly beyond the scope of this communication [9,10,11,12].
- 3DSTE-derived determination of LV strains is validated; therefore, the present study did not aim to validate it again [16,17,18,19].
- The relatively small sample size may raise certain concerns, which we tried to overcome by applying appropriate statistical methods (non-parametric tests, providing median values, etc.).

Conclusions. Only 3DSTE-derived global LV-LS and MAPSE are associated in healthy adults; LV global strains in other directions show no relationship with MAPSE.

## Figures and Tables

**Table 1:** Mitral annular plane systolic excursion and left ventricular volumes and strains in different mitral annular plane systolic excursion groups.

	All Subjects (*n* = 106)	MAPSE ≤ 11 mm (*n* = 15)	11 mm < MAPSE < 17 mm (*n* = 70)	MAPSE ≥ 17 mm (*n* = 21)
LV-EDV (mL)	83.9 ± 21.3 (83.2)	90.9 ± 26.0 (84.4)	80.5 ± 19.4 (80.8)	90.0 ± 21.0 † (90.5)
LV-ESV (mL)	36.0 ± 9.5 (35.1)	39.9 ± 12.2 (38.3)	34.9 ± 8.4 (34.9) *	36.8 ± 10.2 (32.2)
LV-EF (%)	57.4 ± 5.3 (56.5)	56.3 ± 3.5 (56.0)	57.1 ± 5.4 (56.4)	59.1 ± 5.7 (58.1)
LV mass (g)	162.2 ± 31.7 (166.0)	166.9 ± 35.3 (179.0)	161.7 ± 28.9 (169.3)	160.5 ± 37.1 (154)
global LV-RS (%)	25.6 ± 9.9 (24.7)	27.1 ± 7.1 (28.8)	25.1 ± 10.3 (24.2)	26.1 ± 9.9 (23.0)
basal LV-RS (%)	32.2 ± 12.9 (32.7)	34.6 ± 8.2 (36.5)	31.5 ± 12.9 (32.8)	32.6 ± 15.2 (30.2)
midventricular LV-RS (%)	29.8 ± 11.2 (28.1)	30.5 ± 8.7 (28.8)	29.5 ± 12.2 (27.7)	30.5 ± 8.7 (28.1)
apical LV-RS (%)	17.9 ± 9.2 (16.3)	17.5 ± 9.3 (17.8)	17.7 ± 9.4 (16.2)	18.9 ± 8.1 (17.2)
global LV-CS (%)	−27.3 ± 4.8 (−26.6)	−26.8 ± 4.2 (−26.5)	−27.0 ± 4.8 (−26.6)	−28.5 ± 5.2 (−27.0)
basal LV-CS (%)	−25.3 ± 4.6 (−25.6)	−26.8 ± 3.9 (−28.5)	−25.1 ± 4.8 (−25.0)	−25.1 ± 3.8 (−25.6)
midventricular LV-CS (%)	−29.2 ± 5.6 (−28.5)	−28.8 ± 3.9 (−28.7)	−28.8 ± 5.7 (−28.0)	−30.7 ± 6.0 (−30.4)
apical LV-CS (%)	−31.3 ± 10.3 (−30.9)	−28.2 ±11.1 (−28.6)	−31.2 ± 10.0 (−30.9)	−33.8 ± 10.0 (−36.4)
global LV-LS (%)	−16.3 ± 2.4 (−16.3)	−14.9 ± 1.6 (−14.6)	−16.4 ± 2.3 (−16.3) *	−17.0 ± 2.6 (−17.3) *
basal LV-LS (%)	−20.3 ± 4.4 (−20.0)	−19.5 ± 3.6 (−19.6)	−20.2 ± 4.5 (−20.0)	−21.4 ± 4.3 (22.0)
midventricular LV-LS (%)	−13.7 ± 3.5 (−13.5)	−12.5 ± 3.0 (−11.9)	−13.9 ± 3.8 (−13.79	−14.0 ± 2.9 (−13.9)
apical LV-LS (%)	−17.1 ± 5.8 (−16.8)	−15.2 ± 5.4 (−15.5)	−17.4 ± 5.5 (−16.7)	−17.8 ± 6.5 (−17.9)
MAPSE (mm)	14.2 ± 3.0 (14.4)	9.3 ± 1.8 (10.3)	14.0 ± 1.5 (14.1) *	18.4 ± 1.3 (18.1) *†

* *p* < 0.05 vs. MAPSE ≤ 11 mm, † *p* < 0.05 vs. 11 mm < MAPSE < 17 mm. Abbreviations: LV = left ventricular, EDV = end-diastolic volume, ESV = end-systolic volume, EF = ejection fraction, RS = radial strain, CS = circumferential strain, LS = longitudinal strain, MAPSE = mitral annular plane systolic excursion.

**Table 2:** Mitral annular plane systolic excursion and left ventricular volumes and strains in different left ventricular strain groups.

	global LV-RS ≤ 15.7% (*n* = 14)	15.7% < global LV-RS < 35.5% (*n* = 76)	global LV-RS ≥35.5% (*n* = 16)	global LV-CS≤ −22.5% (*n* = 8)	−22.5% < global LV-CS < −32.1% (*n* = 81)	global LV-CS ≥−32.1% (*n* = 17)	global LV-LS ≤ −13.9% (*n* = 18)	−13.9% < global LV-LS < −18.7% (*n* = 72)	global LV-LS ≥−18.7% (*n* = 16)
LV-EDV (mL)	71.9 ± 11.8 (72.1)	84.2 ± 21.9 (84.0)	92.0 ± 21.0 (91.7) *	73.7 ± 12.5 (76.2)	85.4 ± 22.3 (84.5)	81.6 ± 17.8 (82.8)	86.5 ± 24.0 (79.6)	85.1 ± 19.4 (85.6)	75.4 ± 24.1 (78.4)
LV-ESV (mL)	33.9 ± 5.1 (33.0)	36.4 ± 9.5 (35.9)	35.8 ± 12.2 (33.1)	37.0 ± 5.2 (37.5)	37.7 ± 9.4 (34.0)	27.5 ± 6.9 (25.8) †/††	38.6 ± 11.4 (34.9)	36.4 ± 8.9 (36.8)	31.1 ± 8.3 (27.5) ‡/‡‡
LV-EF (%)	53.2 ± 4.6 (52.7)	57.3 ± 4.4 (56.5) *	61.6 ± 6.4 (60.2) */**	49.5 ± 2.9 (50.8)	56.3 ± 3.0 † (56.2)	66.3 ± 4.1 (66.4) †/††	55.3 ± 4.6 (55.2)	57.1 ± 5.1 (56.5)	61.0 ± 5.2 (61.2) ‡/‡‡
LV mass (g)	149.1 ± 29.5 (157.0)	163.5 ± 31.0 (167.5)	167.6 ± 33.7 (179.0)	164.2 ± 25.4 (171.0)	163.3 ± 31.5 (166.0)	155.7 ± 32.2 (162.0)	162.2 ± 33.3 (169.8)	163.9 ± 32.1 (170.0)	154.7 ± 26.6 (156.5)
global LV-RS (%)	11.9 ± 2.9 (12.2)	24.5 ± 5.0 (24.5) *	42.8 ± 7.0 (40.6) */**	18.3 ± 6.5 (20.4)	24.8 ± 8.3† (24.6)	32.7 ± 13.6 (31.0) †/††	24.5 ± 11.7 (20.7)	25.2 ± 9.0 (25.0)	28.4 ± 10.8 (25.7)
global LV-CS (%)	−24.4 ± 4.7 (−23.8)	−27.3 ± 4.4 (−26.7) *	−29.9 ± 5.6 (−28.3) */**	−18.8 ± 2.7 (−18.8)	−26.4 ± 2.5 (−26.4) †	−35.4 ± 2.7 (−34.9) †/††	−26.0 ± 4.5 (−24.8)	−27.1 ± 4.6 (−26.6)	−29.6 ± 4.8 (−28.1) ‡
global LV-LS (%)	−15.7 ± 2.2 (−15.3)	−16.2 ± 2.4 (−16.5)	−16.2 ± 3.4 (−14.9)	−15.8 ± 1.7 (−15.8)	−16.1 ± 2.2 (−16.2)	−17.4 ± 2.9 (−17.9) †	−13.0 ± 1.1 (−13.4)	−16.3 ± 1.3 ‡ (−16.3)	−20.1 ± 1.2 (−19.7) ‡/‡‡
MAPSE (mm)	14.2 ± 2.4 (14.0)	14.1 ± 3.2 (14.4)	14.9 ± 2.6 (14.3)	14.0 ± 2.9 (14.4)	13.9 ± 3.1 (14.0)	15.6 ± 2.5 (15.5)	12.7 ± 3.1 (12.3)	14.3 ± 3.1 (14.3) ‡	15.4 ± 1.9 (15.6) ‡

* *p* < 0.05 vs. global LV-RS ≤ 15.7%, ** *p* < 0.05 vs. 15.7%< global LV-RS < 35.5%, † *p* < 0.05 vs. global LV-CS ≤ 22.5%, †† *p* < 0.05 vs. 22.5% < global LV-CS < 32.1%, ‡ *p* < 0.05 vs. global LV-LS ≤ 13.9%, ‡‡ *p* < 0.05 vs. 13.9% < global LV-LS < 18.6%. Abbreviations: LV = left ventricular, EDV = end-diastolic volume, ESV = end-systolic volume, EF = ejection fraction, RS = radial strain, CS = circumferential strain, LS = longitudinal strain, MAPSE = mitral annular plane systolic excursion.

## Data Availability

The data presented in this study are available on request from the corresponding author.
